# A cowpea severe mosaic virus-based vector simplifies virus-induced gene silencing and foreign protein expression in soybean

**DOI:** 10.1186/s13007-022-00950-7

**Published:** 2022-10-28

**Authors:** Fides Angeli Zaulda, Seung Hyun Yang, Junping Han, Sizolwenkosi Mlotshwa, Anne Dorrance, Feng Qu

**Affiliations:** Department of Plant Pathology, The Ohio State University, Wooster, OH 44691 USA

**Keywords:** Cowpea severe mosaic virus, VIGS, Expression vector, Soybean functional genomics

## Abstract

**Background:**

Soybean gene functions cannot be easily interrogated through transgenic disruption (knock-out) of genes-of-interest, or transgenic overexpression of proteins-of-interest, because soybean transformation is time-consuming and technically challenging. An attractive alternative is to administer transient gene silencing or overexpression with a plant virus-based vector. However, existing virus-induced gene silencing (VIGS) and/or overexpression vectors suitable for soybean have various drawbacks that hinder their widespread adoption.

**Results:**

We describe the development of a new vector based on cowpea severe mosaic virus (CPSMV), a plus-strand RNA virus with its genome divided into two RNA segments, RNA1 and RNA2. This vector, designated FZ, incorporates a cloning site in the RNA2 cDNA, permitting insertion of nonviral sequences. When paired with an optimized RNA1 construct, FZ readily infects both *Nicotiana benthamiana* and soybean. As a result, FZ constructs destined for soybean can be first delivered to *N. benthamiana* in order to propagate the modified viruses to high titers. FZ-based silencing constructs induced robust silencing of phytoene desaturase genes in *N. benthamiana*, multiple soybean accessions, and cowpea. Meanwhile, FZ supported systemic expression of fluorescent proteins mNeonGreen and mCherry in *N. benthamiana* and soybean. Finally, FZ-mediated expression of the Arabidopsis transcription factor MYB75 caused *N. benthamiana* to bear brown leaves and purple, twisted flowers, indicating that MYB75 retained the function of activating anthocyanin synthesis pathways in a different plant.

**Conclusions:**

The new CPSMV-derived FZ vector provides a convenient and versatile soybean functional genomics tool that is expected to accelerate the characterization of soybean genes controlling crucial productivity traits.

**Supplementary Information:**

The online version contains supplementary material available at 10.1186/s13007-022-00950-7.

## Background

With the availability of genome sequences for an increasing number of both model and crop plants, such as Arabidopsis, rice, tomato, wheat, maize, and soybean, many researchers are now focusing their attention on functional genomic characterization of plant genes, especially those playing important roles in the yield and quality of agricultural products, and those conferring resilience to pathogens and abiotic stresses [1–3]. Most functional genomics investigations take the reverse genetics approach that entails knocking out or overexpressing genes-of-interest (GOIs), followed by the examination of phenotypical consequences of these manipulations [4]. Reverse genetics research in some plants that were adopted as early model systems, including Arabidopsis, rice, and tomato, is relatively straightforward for several reasons. First, compared to most crop plants, these plants are relatively easy to transform. Second, thanks to intense, targeted investments and collaborative efforts, relatively saturated mutant libraries are available to be shared by the research community. Additionally, these model plants are relatively easy to grow year-round in greenhouses, making research less prone to seasonal changes in the open fields [5].

By contrast, reverse genetics research on most crops is far more challenging. This is especially true for soybean, the subject of current study [6]. Soybean transformation is inefficient, technically demanding, and routinely takes more than one year to obtain transgenic seed. This, combined with the relatively small numbers of seed each soybean plant produces, makes it extremely challenging to generate mutant seed libraries that cover even 50% of genes. Furthermore, the viability of soybean seed deteriorates very rapidly, meaning that the mutated seed must be replanted every year in order to maintain the mutant libraries, which is an enormous undertaking. Finally, the relatively large size of soybean plants also means that such libraries must be propagated in fields, making the process season-dependent, expensive, labor-intensive, and prone to errors. Therefore, reverse genetics in soybean must rely on alternative approaches.

Virus-induced gene silencing (VIGS) is an alternative functional genomics tool for soybean because it does not involve the generation of inheritable changes. Instead, it uses a virus, most commonly a plus strand (+) RNA virus, that is modified to carry a portion of a plant GOI [7]. Infection of the same plant species with the modified virus triggers a plant defense response known as RNA silencing that targets viral RNA for degradation in a highly sequence-specific manner [7]. Since the virus now carries a host gene fragment, the specific RNA silencing response also generates small interfering RNAs (siRNAs) that target the endogenous mRNA of the plant GOI for degradation, causing the down-regulation (silencing) of the GOI.

Several viruses have been developed into VIGS vectors for use in soybean. A bean pod mottle virus (BPMV)-based VIGS vector was developed more than ten years ago and widely used by the soybean research community [8–11]. Another VIGS vector based on apple latent spherical virus (ALSV) has also been developed and successfully used in select soybean varieties [12–16]. However, both vectors have unique shortcomings. The earliest BPMV VIGS vector relied on in vitro transcribed viral RNAs that must be capped at the 5’ end to be infectious [8]. Such capped transcripts are expensive to prepare. Newer BPMV vectors depended on delivery with the sophisticated particle bombardment equipment [9, 17]. On the other hand, the ALSV-based VIGS vector can only infect a few soybean accessions with sufficient efficacies, greatly limiting its usefulness [13]. Therefore, a more user-friendly and broadly applicable VIGS vector is needed for functional genomic interrogations of soybean genes.

**Fig. 1:**
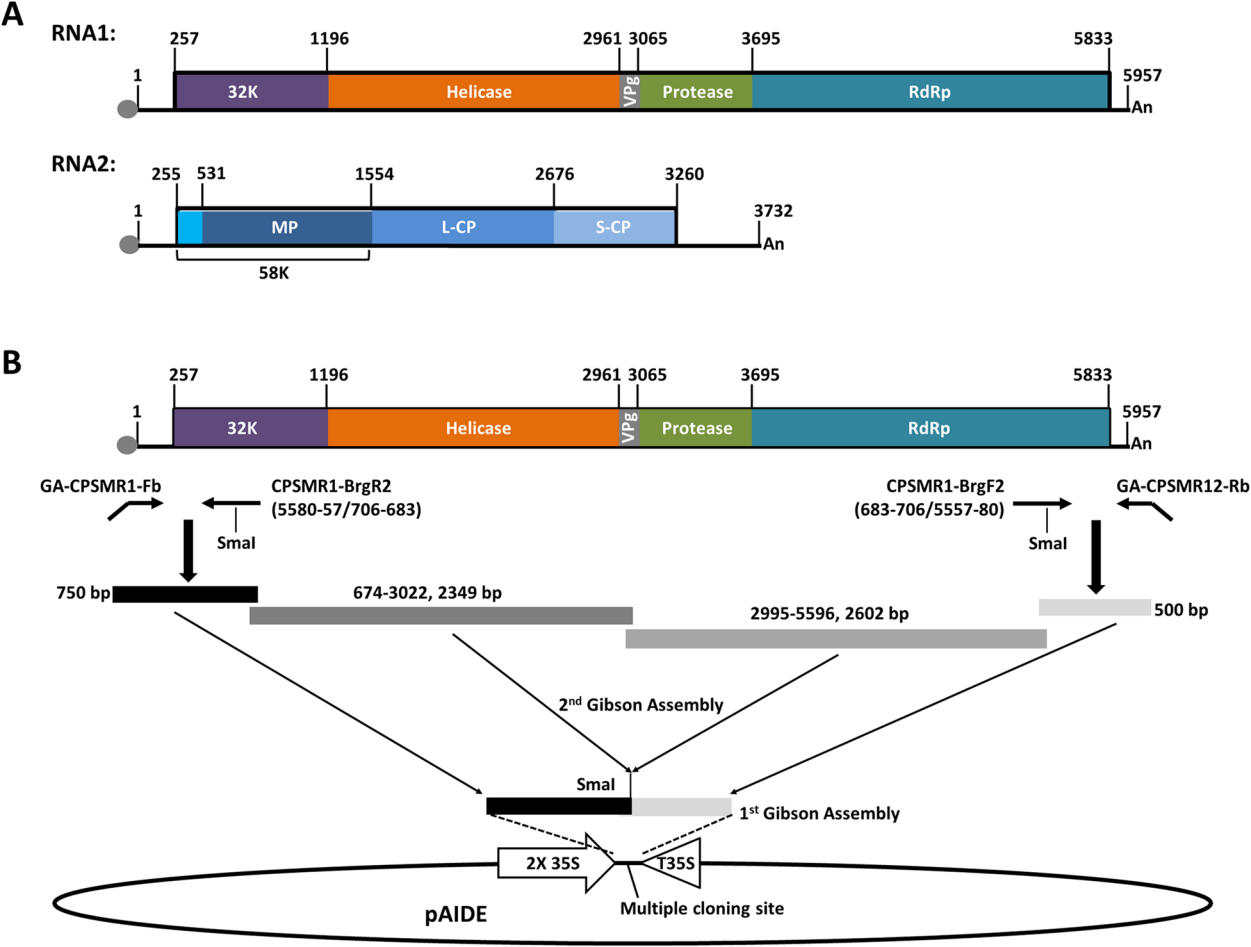
CPSMV genome organization and a two-step strategy for assembling a full-length cDNA clone of CPSMV RNA1. **A** Diagrams of CPSMV RNA1 and 2, the encoded ORFs, and the nt coordinates of various mature proteins. Both genomic RNAs are depicted as thick lines. The small gray oval at the left end denotes the VPg covalently linked to the RNAs, whereas An at the right end denotes a poly-A tail. The numbers above each line denote the nt positions that border various viral proteins, which are in turn depicted as boxes of different colors. **B** Strategy of cloning RNA1 cDNA into pAIDE. The 2X35S promoter and T35S transcriptional terminator in pAIDE ensure the efficient and proper transcription of the inserted RNA1 cDNA. The first cloning step integrates two short terminal fragments, shown as black and light gray boxes respectively, that form a new SmaI site at their junction. The second step then integrates the intervening fragment(s) of the cDNA into the SmaI site

Here we report the development of a new VIGS vector that overcomes the shortcomings of earlier VIGS vectors for soybean. This new vector is based on the cowpea severe mosaic virus (CPSMV), a virus that infects both soybean and the model plant *Nicotiana benthamiana*. CPSMV is a (+) RNA virus belonging to the virus family *Secoviridae,* genus *Comovirus* [18, 19]. Like all comoviruses, the CPSMV genome comprises two (+) RNAs − RNA1 and RNA2, each encoding a single open reading frame (ORF). Upon translation, the two polyproteins are processed into multiple mature, functional proteins by a virus-encoded protease (Pro; Fig. 1A). The RNA1-encoded proteins, including a 32K protein, a helicase, a small genome-linked protein (VPg), Pro, and a viral RNA-dependent RNA polymerase (RdRp), are needed for the replication of both RNA1 and 2, whereas the RNA2-encoded proteins, including a movement protein (MP) and two capsid proteins (L-CP and S-CP), are needed for viral cell-to-cell and systemic movement and particle assembly (Fig. 1A). RNA2 also encodes a 58K protein serving as an accessory protein that presumably recruits replication proteins to RNA2 [17].

In the current study, full-length cDNAs for CPSMV RNA1 and RNA2 were assembled and mobilized into a binary plasmid vector capable of replicating in both *Escherichia coli* and *Agrobacterium tumefaciens*. The resulting infectious clones were delivered into leaves of *N. benthamiana* plants to propagate CPSMV to high titers. CPSMV propagated in this manner was fully infectious in both *N. benthamiana* and soybean. The CPSMV RNA2 cDNA was subsequently modified to give rise to a vector capable of both VIGS and expression of nonviral proteins. This new vector promises to accelerate functional genomic characterization of soybean genes and contribute to the improvement of soybean productivity.

## Results

### Assembly of CPSMV RNA1 and RNA2 infectious clones in the binary vector pAIDE

CPSMV infects both *N. benthamiana* and soybean. Thus, a CPSMV-based VIGS and gene expression vector could be easily propagated in agro-infiltrated *N. benthamiana*, permitting cheap and convenient amplification of inoculums that can be subsequently used to mechanically inoculate soybean. However, to develop a CPSMV-based VIGS and gene expression vector, we first needed to generate infectious clones for both genomic RNAs of CPSMV. To this end, we chose to clone the RNA1 and RNA2 cDNAs into pAIDE, a binary vector modified from pCambia1300 by our lab [17, 20–23]. The modified pAIDE plasmid no longer has the cassette that confers hygromycin resistance in transgenic plants, but acquired a new expression cassette with the duplicated 35S promoter (2X35S) and the 35S terminator (T35S), both originated from cauliflower mosaic virus (Fig. 1B) [17]. The cDNAs of CPSMV RNA1 and RNA2 can then be inserted between 2X35S and T35S, permitting the transcription of CPSMV RNAs in plant cells.

It should be noted that the long poly-A tail at the 3′ ends of CPSMV RNA1 and 2 play critical role in the translation of viral proteins, as well as in viral genome replication, hence must be part of infectious cDNA clones. However, a reverse primer with a long stretch of T residues made long-range PCR very challenging. To address this problem, a stepwise strategy was devised to clone the CPSMV cDNAs. As shown in Fig. 1B, the first cloning step entailed the generation of two relatively short PCR fragments (< 1 kb) corresponding to two termini of the full-length viral cDNAs, bridged to each other with a unique restriction enzyme site (SmaI). As a result, the long poly-dT tract was relatively easily incorporated into the short 3′ fragment with a poly-dT-containing reverse primer (GA-CPSMR12-Rb; Fig. 1B; Additional file 1: Table S1). The second cloning step then inserted the middle portion of the viral cDNAs. In the case of RNA1 cDNA, the middle portion was assembled with two overlapping fragments (Fig. 1B).

**Fig. 2:**
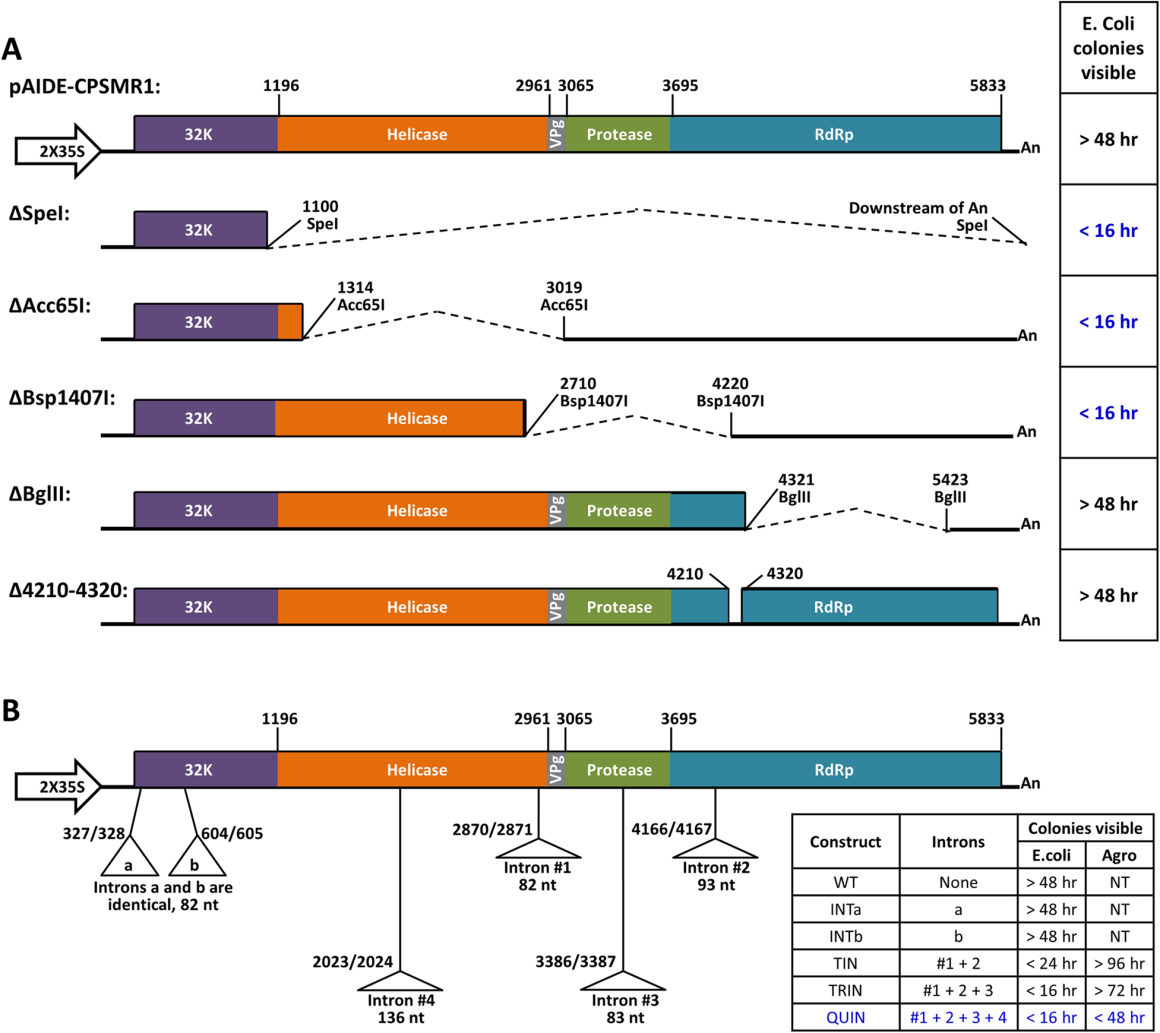
Mapping and modifying regions of CPSMV RNA1 cDNA conferring bacterial toxicity. **A** Mapping regions in RNA1 cDNA conferring toxicity to *E. coli*. Diagrams of five deletion mutants are presented, with the deleted region denoted with dashed lines. The one-column table to the right summarizes the effect of the deletions on *E. coli* growth rate, which correlates inversely with the toxicity of RNA1 cDNA. **B** Positions of CPSMV RNA1 cDNA into which various introns have been inserted in order to improve the replication efficiency of the corresponding constructs. All intron-containing constructs are listed in the table to the right, along with the intron(s) they possess, and their impacts on *E. coli* and *A. tumefaciens* growth rates. *hr* hours, *WT* wildtype, *NT* not tested

**Fig. 3:**
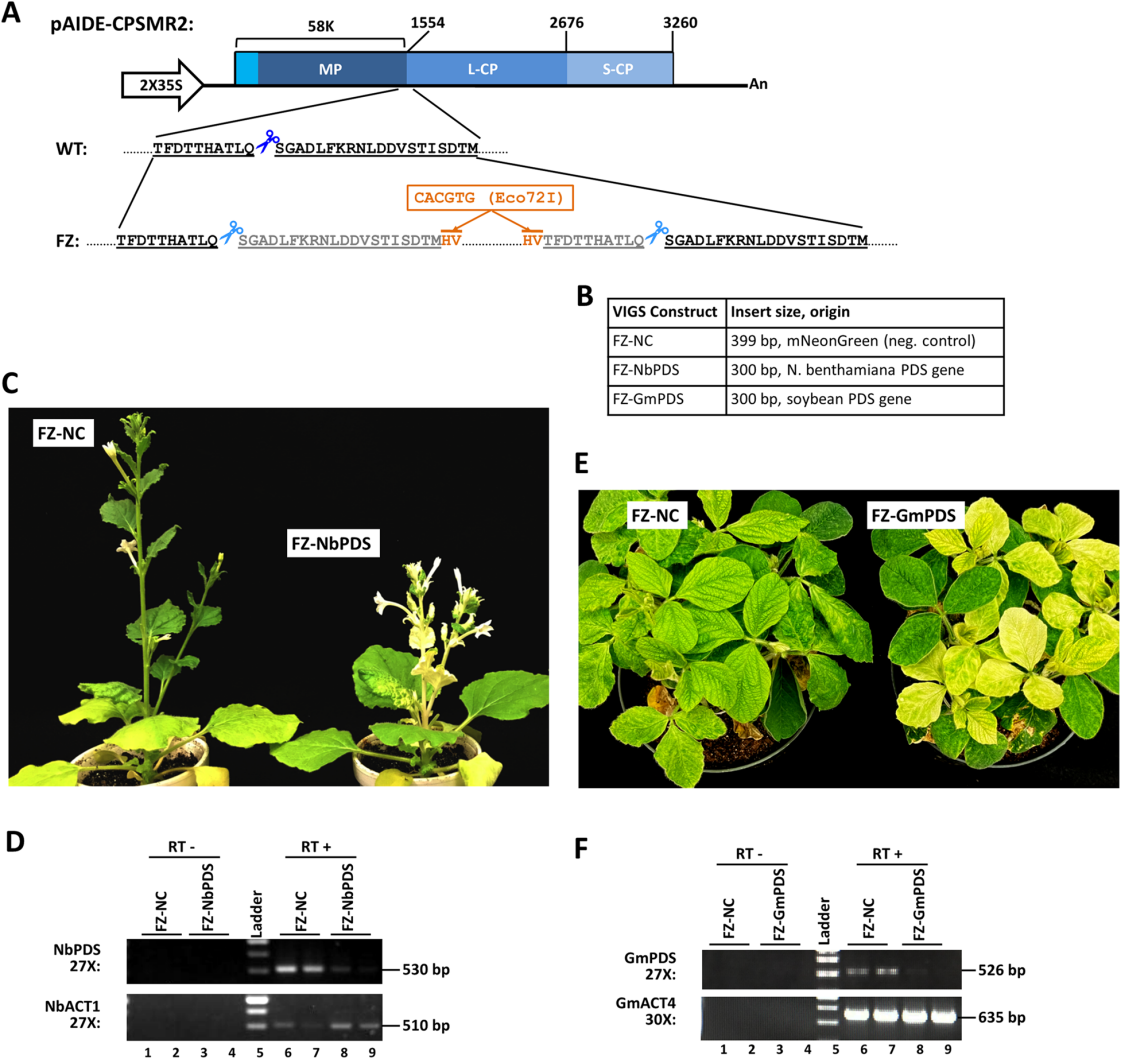
Engineering CPSMV RNA2 cDNA into a vector for VIGS and nonviral gene expression. **A** Diagram of the RNA2 cDNA under control of the 2X35S promoter, with the amino acid sequences flanking the protease processing site between MP and L-CP shown immediately below (10 aa residues before the processing site, and 20 after). The processing site is denoted with a pair of dark blue scissors. The bottom row shows the FZ constructs with the protease processing site duplicated, and two Eco72I sites introduced for future insertion of GOI fragments. Note that the two protease processing sites are now denoted with light blue scissors, and the duplicated aa residues in gray letters, both reflecting the fact that the nt sequence encoding the duplicated aa residues has been codon-shuffled to minimize nt sequence identity, hence the chance of recombination. **B** A table listing one control construct (FZ-NC), and two VIGS constructs (FZ-NbPDS and FZ-GmPDS), along with their insert sizes. **C** Photobleaching in *N. benthamiana* due to FZ-NbPDS-induced VIGS of NbPDS. **D** Verification of reduced NbPDS mRNA levels with sqRT-PCR. RT-: control reaction without reverse transcriptase. **E** Photobleaching in soybean (accession Williams 82) due to FZ-GmPDS-induced VIGS of GmPDS. **F** Verification of reduced GmPDS mRNA levels with sqRT-PCR

Despite initial setbacks, we eventually succeeded in obtaining the cDNA constructs for both RNA1 and RNA2 of CPSMV, with their sequences fully verified through Sanger sequencing. These constructs were named pAIDE-CPSMR1 and pAIDE-CPSMR2, respectively (Figs. 2 and 3). Notably, while pAIDE-CPSMR2 replicated robustly in both *E. coli* and *A. tumefaciens*, pAIDE-CPSMR1 replicated extremely slowly in *E. coli*, and failed to form any visible colonies when transformed into *A. tumefaciens*. These results suggested that similar to many other (+) RNA viruses [24], the cDNA of CPSMV RNA1 might direct the production of protein(s) or peptide(s) toxic to both bacteria, and that additional manipulations were needed to alleviate the toxicity of the construct containing RNA1 cDNA.

### Stepwise attenuation of the toxicity of pAIDE-CPSMR1

Previous studies by others showed that plasmids harboring cDNAs of (+) RNA viruses were often toxic to hosting bacteria, but this toxicity could be neutralized by inserting introns into the protein-coding region(s) of these cDNAs [24–26]. This is likely due to the inability of prokaryotes such as *E. coli* or *A. tumefaciens* to process introns, as introns are only present in eukaryotic genes. To determine whether introns could be used to mitigate the toxicity of pAIDE-CPSMR1, we first attempted to map the regions in CPSMV RNA1 cDNA most likely to cause bacterial toxicity by generating a series of deletions within the RNA1 cDNA. The resulting constructs were tested in *E.coli*, using the time needed to produce visible colonies as an inverse indicator of toxicity. Figure 2A lists some of the deletions, with their effects on *E. coli* growth shown to the right. Deleting most of the RNA1 cDNA (ΔSpeI, from nt position 1,100 to the very 3’ end) completely abrogated the toxicity, restoring normal growth to *E.coli* (colonies became visible in less than 16 h). Two smaller deletions, ΔAcc65I and ΔBsp1407I, between positions 1314–3069 (1706 bp) and 2710–4220 (1511 bp) respectively, restored normal *E. coli* growth as well. However, a deletion encompassing positions 4321–5423 (ΔBglII, 1103 bp), as well as a small in-frame deletion spanning positions 4210–4320 (111 bp), failed to restore normal *E. coli* growth. Thus, the 2710-to-4320 region appears to be responsible for most of the bacterial toxicity conferred by CPSMV RNA1 cDNA.

We then began to insert introns into various positions of the RNA1 cDNA. Since RNA1 encodes a single ORF, it is possible that introns inserted near its 5′ end, by disrupting the translation of the entire polyprotein near its N-terminus, could exert a strong mitigating effect on its toxicity. We hence chose two locations near the 5′ end of RNA1 cDNA for initial intron insertion attempts. These locations are nt positions 327/328, and 604/605, both harboring the consensus splicing border motif AG/GT(T corresponds to U in RNA) (Fig. 2B). The introns a and b were identical, being derived from an 82-nt intron of *AtPDS3*. They were inserted into RNA1 cDNA separately, resulting in two constructs—INTa and INTb. Surprisingly, neither of them restored normal growth to transformed *E. coli*. Therefore, introns inserted at these two positions were ineffective at alleviating the toxicity of RNA1 cDNA.

Given the failure of INTa and INTb, we next turned to the region deleted in ΔBsp1407I (nt positions 2710–4220; Fig. 2A). Two introns of 82 and 93 nt in size, both derived from *AtPDS3*, were then simultaneously inserted into two locations in RNA1 cDNA—2870/2871 and 4166/4167 (Fig. 2B, Intron #1 and #2). The resulting construct was named as TIN (for two introns). Upon transforming into *E. coli*, TIN caused substantially faster bacterial growth, indicating a dramatic reduction of plasmid toxicity in *E. coli* (Fig. 2B). *A. tumefaciens* was also able to survive from TIN, but the colonies grew extremely slowly, becoming visible only after 4 days of growth at 28 °C. This indicated to us that CPSMV RNA1 cDNA harbored additional region(s) that conferred specific toxicity to *A. tumefaciens*.

To further attenuate the toxicity of CPSMV RNA1 cDNA, we next created two more constructs, TRIN and QUIN, that contained three and four introns, respectively (Fig. 2B). Specifically, TRIN was a TIN derivative with an additional 83-nt intron (Intron #3) at the position 3386/3387; whereas QUIN had a fourth intron at the position 2023/2024 (Fig. 2B). While TRIN showed a modest improvement over TIN in both *E. coli* and *A tumefaciens*, QUIN propagated in both bacteria to levels equivalent to insert-free pAIDE (Fig. 2B). More importantly, when paired with pAIDE-CPSMR2 and delivered into *N. benthamiana* with agro-infiltration, QUIN initiated systemic, symptomatic CPSMV infections in all plants (see below). Therefore, inserting four introns at multiple positions of CPSMV RNA1 cDNA caused a near complete neutralization of pAIDE-CPSMR1 toxicity in both *E. coli* and *A. tumefaciens*. As a result, QUIN was used in subsequent experiments as the provider of CPSMV RNA1.

### A CPSMV-based VIGS vector induces robust silencing of *PDS *genes in *N. benthamiana* and soybean

To develop a VIGS vector using CPSMV cDNA clones, we next modified pAIDE-CPSMR2. Specific modifications included: (i) the protease processing site between MP and L-CP were duplicated by repeating the 20 aa residues downstream, and then 10 aa residues upstream, of the processing site (gray letters in Fig. 3A); (ii) the nt sequence of the duplicated aa residues were extensively codon-shuffled to minimize the nt-level identity while maintaining the aa sequence, hence reducing the chance of homologous recombination (see Additional file 2: Table S2, bottom row for sequence details); (iii) two Eco72I sites were inserted within the duplicated processing site to permit the cloning of nonviral sequences (orange letters, lines, and box in Fig. 3A); and (iv) a 300 bp fragment of *N. benthamiana PDS* (*NbPDS*) was inserted between the Eco72I sites to permit the testing of VIGS in *N. benthamiana*. The resulting construct was designated FZ-NbPDS (the vector itself was named as FZ). Note that Gibson Assembly cloning was used to incorporate all VIGS and gene expression inserts to ensure their proper orientation.

We then mixed *A. tumefaciens* strains containing QUIN (RNA1) and FZ-NbPDS (RNA2), and delivered them into young *N. benthamiana* leaves through agro-infiltration. FZ-NC, a construct containing a 399-nt non-plant, non-virus insert (partial coding sequence of mNeonGreen, a green fluorescent protein) was included as a negative control (Fig. 3B). As shown in Fig. 3C, *N. benthamiana* plants treated with FZ-NbPDS (plus QUIN) exhibited extensive photobleaching that was absent in FZ-NC (plus QUIN) plants by 21 days after agro-infiltration. Consistent with the photobleaching phenotype, the *NbPDS* mRNA levels as determined by semi-quantitative (sq) RT-PCR decreased substantially (Fig. 3D). Together these results demonstrated FZ as a robust VIGS vector in *N. benthamiana*.

We next tested the effectiveness of FZ as a VIGS vector in soybean. To do this, we inserted a 300 bp fragment of the *GmPDS1* (*Glycine max PDS1*) gene into FZ, resulting in FZ-GmPDS. Since soybean is recalcitrant to agro-infiltration, the FZ-GmPDS (plus QUIN) was first propagated in agro-infiltrated *N. benthamiana* plants. The symptomatic *N. benthamiana* leaves harvested at 14 days after agro-infiltration were ground and used to inoculate young soybean plants (accession Williams 82). Figure 3E shows soybean plants at 21 days after inoculation. FZ-GmPDS infection consistently caused widespread photobleaching that was absent in the control plants inoculated with FZ-NC. The bleaching results were correlated with a near complete loss of GmPDS1 mRNA, as determined with sqRT-PCR (Fig. 3F). Therefore, the FZ VIGS vector was also highly efficient at silencing soybean genes.

### *PDS* genes in multiple soybean accessions, as well as cowpea, are consistently silenced by FZ-GmPDS

**Fig. 4:**
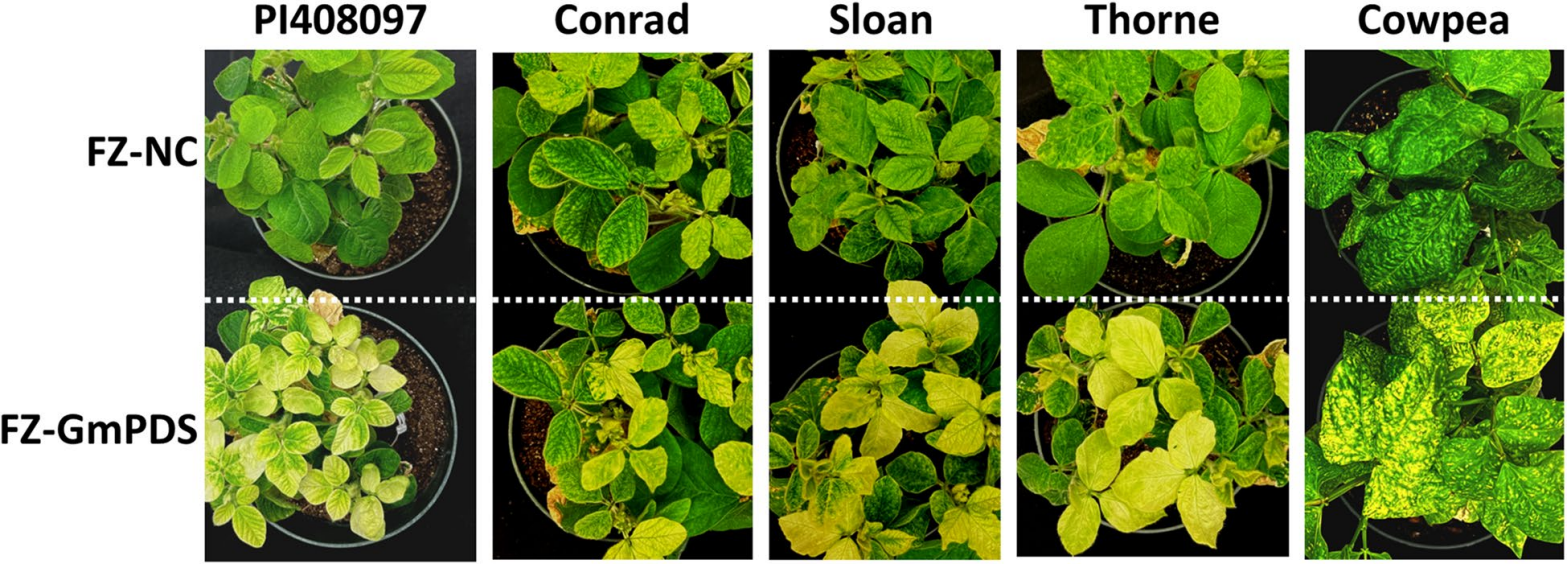
Photobleaching in four additional accessions of soybean—PI408097, Conrad, Sloan, and Thorne, and in cowpea, due to FZ-GmPDS-induced VIGS of the *PDS* gene. The pots shown on the top contained control plants inoculated with the FZ-NC control construct. The images were collected at 4 weeks post inoculation

A previous study [13] found that an ALSV-based VIGS vector was effective in only a few plant introduction (PI) lines of soybean (e.g., PI567301B), but ineffective in most cultivated accessions, such as Conrad, Sloan, and Thorne. To assess how effective the new FZ vector was in these soybean accessions, FZ-GmPDS was tested in four additional soybean lines: PI408097, Conrad, Sloan, and Thorne. Since the *GmPDS* insert in FZ-GmPDS shared more than 95% sequence identity with a cowpea *PDS* gene, we also included cowpea (*Vigna unguiculata*, variety California Black-Eye) in this set of experiments. As shown in Fig. 4, all of the plants inoculated with FZ-GmPDS, but none inoculated with the FZ-NC control, showed extensive photobleaching in top young leaves by three weeks post inoculation. Furthermore, photobleaching persisted until at least six weeks post inoculation, when the experiments were terminated. Therefore, this CPSMV-based vector achieved robust VIGS in all soybean accessions tested, and was also active in cowpea.

### FZ supports strong and lasting expression of fluorescent proteins in both *N. benthamiana* and soybean

**Fig. 5:**
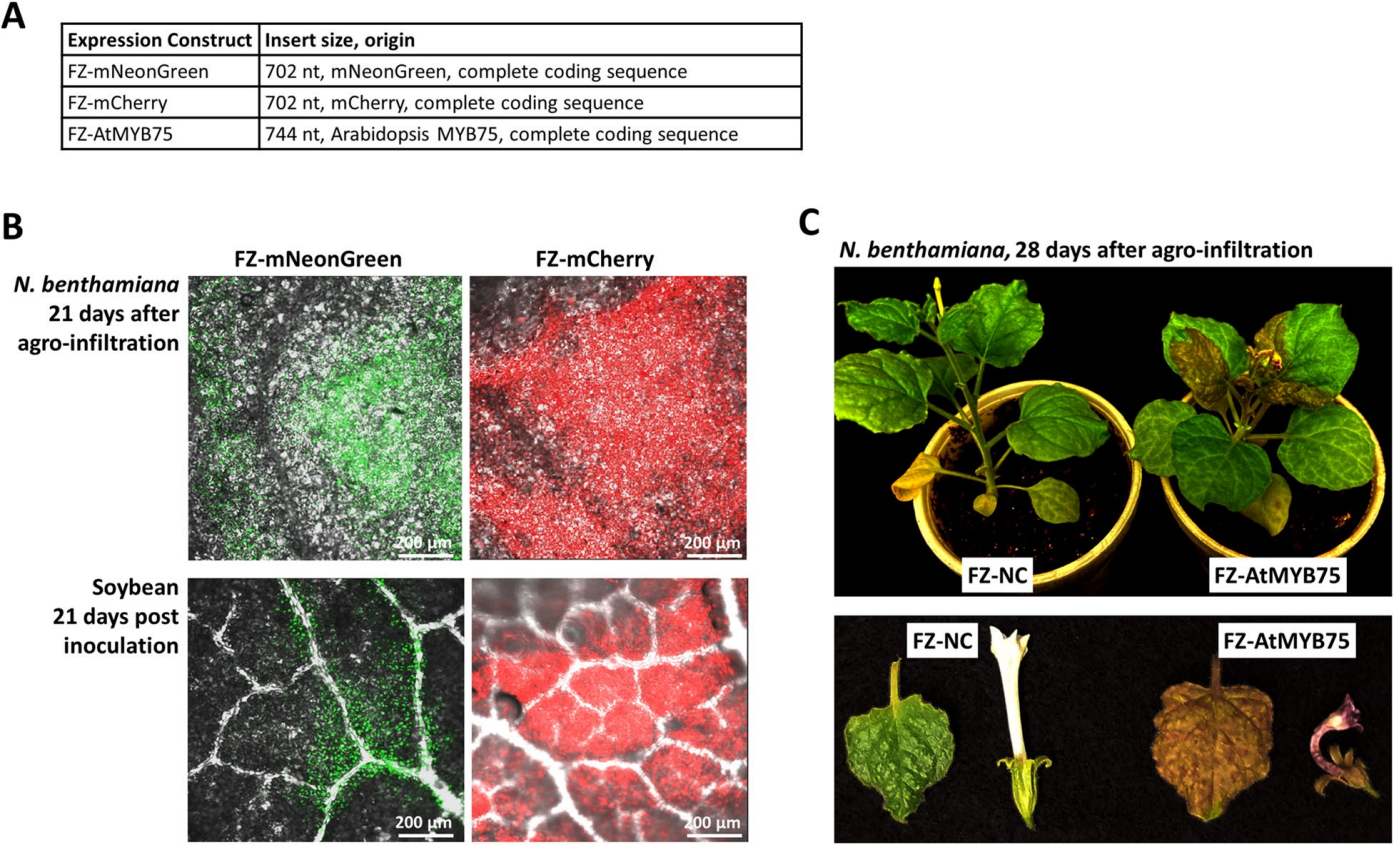
FZ-mediated expression of nonviral proteins in *N. benthamiana* and soybean. **A** Three different FZ constructs harboring coding sequences of mNeonGreen, mCherry, and AtMYB75, respectively. **B** FZ-mediated expression of mNeonGreen and mCherry in *N. benthamiana* and soybean. Shown are sections excised from systemically infected leaves, at 21 days after agro-infiltration (*N. benthamiana*) or rub inoculation (soybean). C. Purple leaves and flowers of *N. benthamiana* plants agro-infiltrated with FZ-AtMYB75. The top panel shows the intact plants, whereas the bottom panel shows the detached leaves and flowers for higher resolution

We next assessed whether FZ was suitable for expressing nonviral proteins. The coding sequences of the green-fluorescent protein mNeonGreen, and the red-fluorescent protein mCherry, were cloned into FZ, leading to constructs FZ-mNeonGreen and FZ-mCherry. Note that these inserts were both 702 nt (Fig. 5A), approximately twice as large as the NbPDS, GmPDS, or NC inserts. These constructs, along with the QUIN construct encoding CPSMV RNA1, were transformed into *Agrobacterium* and introduced into *N. benthamiana* leaves with agro-infiltration. Signs of systemic infection became apparent in *N. benthamiana* plants 7–8 days later. Accordingly, green and red fluorescence were observed in the top young leaves of the respective plants at about the same time. Note that the mNeoGreen and mCherry fluorescence differ in excitation wavelengths (488 vs 587 nm, respectively). As a result, the FZ-mNeonGreen-infected leaves served as the negative control for those infected with FZ-mCherry, and vice versa (not shown). Consistent with the stable retention of intact mNeonGreen and mCherry coding sequences, fluorescent protein expression persisted until at least 21 days after agro-infiltration (Fig. 5B). These results indicated that the FZ vector was a potent vector for expressing nonviral proteins in *N. benthamiana*.

For inoculating soybean plants, systemic *N. benthamiana* leaves were collected 14 days after agro-infiltration, homogenized, and mechanically applied to young soybean leaves. Consistently, the inoculated soybean plants began to show signs of systemic CPSMV infection 6–8 days post inoculation, and the systemic symptoms became easily observable in all inoculated soybean plants by 14 days. The systemically infected top young leaves were also inspected with confocal microscopy and found to contain large green and red fluorescent leaf areas that were easily detectable (Fig. 5B, bottom) until at least 21 days post inoculation. Together these results demonstrated that the FZ vector was also a potent vector for expressing nonviral proteins in *N. benthamiana* and soybean.

### MYB75 of Arabidopsis expressed from FZ is functionally active in *N. benthamiana*

To test whether nonviral proteins expressed using the FZ vector retained their original function, The coding sequence of the MYB75 transcription factor of Arabidopsis was cloned into FZ. MYB75, also known as PAP1 (for PRODUCTION OF ANTHOCYANIN PIGMENT 1), activates the expression of genes in the metabolic pathways that synthesize multiple phenylpropanoid compounds, among them anthocyanins [27–29]. Overaccumulation of anthocyanins causes plant leaves and flowers to display shades of purple color, providing an easily tractable phenotype for MYB75 overexpression. Indeed, a faint purple color began to appear at 5 days after agro-infiltration on the *N. benthamiana* leaves infiltrated with FZ-AtMYB75 (plus QUIN). More prominently, the top young leaves took an intensely purple appearance starting 14 days after agro-infiltration (Fig. 5C). Additionally, flowers that emerged from these plants also became intensely purple (instead of white as in control plants). Therefore, FZ-expressed AtMYB75 retained its function as an activator of anthocyanin synthesis in the heterologous *N. benthamiana*. Interestingly, the flower pedals, which in *N. benthamiana* plants are fused to form a long, straight tube, became bent and twisted in FZ-AtMYB75-infected plants. This phenotype has not been reported in Arabidopsis, thus may suggest additional novel functions for AtMYB75.

## Discussion

The current study achieved several goals. First, infectious cDNA clones for both genomic RNAs of CPSMV were successfully constructed in a binary vector capable of replication in both *E. coli* and *A. tumefaciens*, allowing us to initiate CPSMV infections through the cost-saving agro-infiltration approach. Second, a CPSMV RNA2-based vector was successfully engineered by modifying the protease processing site separating the MP and L-CP coding regions, permitting in-frame insertion of nonviral sequences. Third, potent VIGS, as well as consistent foreign protein expression, was achieved with this FZ vector, in both *N. benthamiana* and soybean plants. This vector represents a valuable addition to the toolbox for soybean functional genomics studies.

The considerable difficulty in obtaining a stable construct harboring cDNA of CPSMV RNA1, while frustrating, is not unique to CPSMV. Indeed, numerous previous reports have documented the apparent toxicity exerted by viral cDNAs to bacteria including *E. coli* and *A. tumefaciens* [24, 30–32]. It was thought that some of the virus-encoded proteins, especially those involved in viral genome replication, may contain domains that could form growth-arresting protein aggregates in bacteria. Additionally, long viral cDNA fragments might contain cryptic promoter elements that could facilitate the transcription of partial or full-length viral RNA, providing mRNA for the translation of toxic protein domains [30]. Consistent with this idea, we found that the bacterial toxicity exerted by CPSMV RNA1 mapped to multiple regions. Furthermore, it appears that separate regions controlled toxicities to *E. coli* and *A. tumefaciens*. Previous studies also demonstrated that inserting introns at various locations of viral cDNA could alleviate the toxicity, presumably by disrupting the protein translation and/or the integrity of the cryptic promoters [24–26]. These introns are not removable in bacteria, but can be readily spliced out in eukaryotic cells, returning the viral RNA to its original sequence. In our investigations, the bacterial toxicity of CPSMV RNA1 cDNA was successfully overcome by incorporating introns at four different locations, within the helicase and RdRp coding sequences (Fig. 2B).

The fact that the FZ vector was robustly functional in both *N. benthamiana* and soybean offers many advantages when compared to other virus-based vectors developed previously for use in soybean. These earlier vectors include some based on BPMV, and others based on ALSV [8, 9, 12, 16]. Our lab has had experiences with both vector systems[13, 33]. Despite their widespread use, the BPMV vectors has a major drawback: BPMV does not infect *N. benthamiana*. As a result, the inoculum of BPMV vectors must be either in vitro transcribed viral RNAs, which due to the need for the 5′ cap is very expensive to produce, or cDNAs that require pricy particle bombardment equipment for delivery into plants. One also must contend with the time-consuming process of particle bombardment, taking approximately 10 min to inoculate a single seedling. On the other hand, although ALSV vectors could be propagated in agro-infiltrated *N. benthamiana* plants [13], they induced robust silencing only in very few PI lines. By contrast, our FZ vector can be easily and cost-effectively propagated in *N. benthamiana*, and it infects all soybean accessions and cultivars we tested. Thus, FZ represents a superior choice for soybean functional genomics applications.

## Conclusions

We have succeeded in developing a new VIGS and gene expression vector for soybean that is broadly applicable, easy and economical to use, and yields robust and consistent outcomes. Adoption of this vector by fellow soybean researchers is expected to accelerate functional characterization of soybean genes, laying the foundation for informed improvement of soybean germplasms, contributing to higher soybean yield.

## Methods

### Generation of full-length CPSMV infectious clones

The original CPSMV stock (strain CB) was kindly provided by Dr. George Bruening of University of California-Davis. The virus was used to infect *N. benthamiana* plants, from which total RNA was extracted. The total RNA, containing both CPSMV RNAs, was used to generate viral cDNAs through reverse transcription (RT), with a poly-dT-containing oligo (GA-polyT_f_4i; Additional file 1: Table S1) as the primer. The RT reaction was carried out using a First Strand cDNA Synthesis Kit (Thermo Fisher) following the manufacturer’s instructions. To subsequently generate double stranded CPSMV cDNA with PCR, multiple overlapping pairs of primers (Additional file 1: Table S1) were designed based on the NCBI reference sequences NC_003545.1 (RNA1) and NC_003544.1 (RNA2).

The resulting cDNA fragments were sequenced to verify the complete sequences of the RNA1 and RNA2 of this CPSMV isolate. The size of RNA1 was identical to NC_003545.1 [5,957 nucleotides (nt) excluding the poly-A tail], but its sequence contained eight single nt differences (G287A, U2569C, U3377C, A3451G, U3548C, A5208G, G5278U, and U5824C), resulting in two amino acid (aa) changes in the RNA1 polyprotein (S1098P and K1651R). Both the size and the sequence of RNA2 were identical to NC_003544.1 (3,732 nt excluding the poly-A tail) [18, 19].

To assemble full length cDNAs of CPSMV RNA1 and RNA2, we first cloned short fragments corresponding to their 5′ and 3′ termini into the binary vector pAIDE (a derivative of pCambia1300) [17, 20], connecting the two termini with unique restriction enzymes sites. Figure 1B illustrates the specifics with RNA1 cDNA as the example. Briefly, the terminal fragments of RNA1 cDNA, 750 and 500 base pairs (bp) in size, were PCR-amplified with primer pairs GA-CPSMR1-Fb/CPSMR1-BrgR2, and GA-CPSMR12-Rb/CPSMR1-BrgF2 (Additional file 1: Table S1). The 5′ end of the 5′ fragment, and the 3′ end of the 3′ fragment, respectively, shared sequence identities of 17 base pairs (bp) each with the flanking vector sequences. Furthermore, the 3′ end of the 5′ fragment and the 5′ end of the 3′ fragment shared a 23-bp sequence identity including an embedded SmaI site (Fig. 1B). These overlapping regions permitted a three-party ligation through Gibson Assembly cloning (NEBuilder Assembly kit, New England Biolabs), yielding the intermediary construct pAIDE-CPSMR1-FbRb2.

Subsequently, the middle region spanning nt positions 705-5557 of RNA1 was PCR-amplified as two overlapping fragments, using primer pairs CPSMR1-674F/CPSMR1-3022R, and CPSMR1-2995F/CPSMR1-5596R (Additional file 1: Table S1). These two fragments, 2349 and 2602 bp respectively, overlapped for 27 bp. They were cloned into pAIDE-CPSMR1-FbRb2, digested with SmaI, through three-party Gibson Assembly. Note that while the cloning was eventually successful after repeated attempts, the resulting *E. coli* colonies were extremely small, suggesting that the full-length CPSMV RNA1 cDNA insert was toxic to *E. coli.* One clone was sequence-verified for the entire insert, and designated pAIDE-CPSMR1. As will be described in the Results section, this clone had to be further optimized to improve its viability and stability in *E. coli* as well as in *A. tumefaciens*.

A similar strategy was used to generate a CPSMV RNA2 cDNA construct. First, two RNA2 terminal fragments were PCR-amplified with primer pairs GA-CPSMR2-Fb/CPSMR2-BrgR2, and GA-CPSMR12-Rb/CPSMR2-BrgF2 (Additional file 1: Table S1). These two fragments, 710 and 560 bp, respectively, were cloned into pAIDE with Gibson Assembly by virtue of their terminal sequence identities. A larger middle fragment spanning nt positions of 631-3243 was PCR-amplified with the primer pair CPSMR2-631F/CPSMR2-3243R, and inserted between the terminal fragments to obtain the plasmid pAIDE-CPSMR2. The identity of the entire insert was verified with Sanger sequencing.

### Insertion of introns in the CPSMV RNA1 cDNA

Sequences of introns (see Additional file 2: Table S2 for details) were incorporated into the sequence of CPSMV RNA1 cDNA at several sites with the AG/GT splicing border motifs (Fig. 2), and the resulting sequences were used for synthesizing gBlock fragments (Integrated DNA Technologies) that were subsequently inserted into pAIDE-CPSMR1 with Gibson Assembly. The sequences of the gBlock fragments are listed in Additional file 2: Table S2. Introns a, b, and #1 are identical and were derived from the Arabidopsis *PHYTOENE DESATURASE* (*AtPDS3*) gene (At4G14210). Intron #2 is a different intron of *AtPDS3*. Introns #3 and 4 were both derived from the Arabidopsis *DICER-LIKE 4* (*AtDCL4*) gene.

### Generation of the FZ vector by modifying pAIDE-CPSMR2

A gBlock fragment, FZvec-ML-NbPDS (Additional file 2: Table S2), was custom-synthesized, and used to replace the AflII-Eco72I fragment of CPSMV RNA2 cDNA with Gibson Assembly cloning. Note that this cloning step eliminated the original Eco72I site within the RNA2 cDNA, permitting us to use the new Eco72I sites flanking the NbPDS insert for subsequent cloning of other inserts. The sequence identity of the inserted fragments has been verified with Sanger sequencing.

### *Agrobacterium* infiltration (agro-infiltration)

All constructs subject to infectivity tests were transformed into electrocompetent *A. tumefaciens* strain C58C1 via electroporation using the AGR setting on the Bio-Rad Micropulser Electroporator [21, 34]. Briefly, 5 µl of the plasmid DNA was mixed with 40 µl electro-competent Agrobacterium cells and maintained on ice until electroporation. After electroporation, 900 µl of SOB media was added and the suspension was incubated at 28 °C for one hour. Selection was carried out on solid Terrific Broth (TB) media containing rifampicin, gentamycin, and kanamycin. Successful introduction of the plasmid was confirmed using colony PCR. A single colony confirmed to have the desired plasmid was used to inoculate 3 ml TB liquid media with the same antibiotics, and incubated overnight at 28 °C. The culture was diluted 1:100 with fresh TB liquid media and incubated under the same conditions for another night. The second culture was centrifuged at 4000 rpm for 20 min, and resuspended in agroinfiltration buffer (10 mM MgCl_2_, 10 mM MES, and 100 µM acetosyringone). All suspensions were diluted to OD_600_ = 1 and incubated at 28 °C for 3 h. *Agrobacterium* suspensions were then mixed and introduced into leaves of young *N. bethamiana* plants via a small wound, using a needleless syringe.

### Confocal microscopy

At 1–3 weeks post inoculation/agro-infiltration, leaf discs were collected from the plants. Confocal microscopy was performed at the Molecular and Cellular Imaging Center (MCIC), the Ohio Agricultural Research and Development Center, using the Leica TCS SP6 confocal scanning microscope. Sequential excitation at 488 nm and 587 nm was provided by argon and helium–neon 543 lasers, respectively; and the corresponding mNeonGreen and mCherry fluorescent images were captured with the respective filter sets provided with the microscope [17, 20, 22, 35].

### Semi-quantitative (sq) RT-PCR

Total RNA was extracted from the systemically infected leaves of *N. benthamiana* or soybean using the Zymo RNA Extraction kit. A DNase treatment step was always included to eliminate potential contamination of plant genomic DNA. The quality of the RNA samples was assessed first with a NanoDrop spectrometer, followed by agarose gel electrophoresis to determine the RNA integrity. For sqRT-PCR, an equal amount (usually 1 µg) of RNA was used for all samples in the same experimental group, and the 1st strand cDNA synthesis was primed with a poly-dT primer. At the PCR step, a pair of soybean actin 1 primers were used to amplify a cDNA fragment that serves as a control to ensure that cDNA synthesis in all samples was initiated with an equivalent amount of mRNA. Various primer pairs (Additional file 1: Table S1) that amplify either viral (e.g., CPSMV RNA2) or plant (e.g., GmPDS1) cDNAs were used to assess the levels of the corresponding mRNAs.

## Supplementary Information

**Additional file 1: Table S1. **Primers used in the current study.

**Additional file 2: Table S2. **Sequences of gBlock fragments used in this study.

## Data Availability

The QUIN and FZ-NbPDS plasmids will be available through Addgene (https://www.addgene.org/) prior to the official date of publication.

## Abbreviations

VIGS: Virus-induced gene silencing
CPSMV: Cowpea severe mosaic virus
RNA: Ribonucleic acid
cDNA: Complementary DNA
sqRT-PCR: Semi-quantitative reverse transcription–polymerase chain reaction
