# Supplementary material for: A cowpea severe mosaic virus-based vector simplifies virus-induced gene silencing and foreign protein expression in soybean
Source: Plant Methods. 2022 Oct 28;18:116. doi: 10.1186/s13007-022-00950-7 (PMC9617382; doi:10.1186/s13007-022-00950-7)
Supplement: Supplementary file 1 — Additional file 1: Table S1. Primers used in the current study. [file 13007_2022_950_MOESM1_ESM.docx]

**Table S1.** Primers used in the current study

| **Name** | **Sequence (5’ to 3’)** | **Purpose** |
| --- | --- | --- |
| GA-polyT_f_4i | ACTGGTGATTTTTGCGGACTCTA GATTTTTTTTTTTTTTTTTTTTTTTTT | cDNA synthesis of poly-adenylated RNA (including CPSMV RNAs) |
| GA-CPSMR1-1Fb | TTCATTTGGAGAGGACCGGTTTATTAA AATTTTCAAGAGAAGATTTTGA | Generation of full-length CPSMV RNA1 cDNA with PCR |
| GA-CPSMR12-Rb | GGTGATTTTTGCGGACTAGTTTTTTTTTT TTTTTTTTTTTTTTTAAAAG | Generation of full-length CPSMV RNA1 and RNA2 cDNAs with PCR |
| CPSMR1-BrgF2 | TCAGGACTTGCTGACTGGATTCCC GGGAGGACTGCCGACTCATGGTTG | Stepwise generation of the full-length cDNA of CPSMV RNA1 |
| CPSMR1-BrgR2 | CAACCATGAGTCGGCAGTCCTCCC GGGAATCCAGTCAGCAAGTCCTGA | Stepwise generation of the full-length cDNA of CPSMV RNA1 |
| CPSMR1-522F | GCACAAAGATTTCAAACTCAAAGG | PCR, sequencing of CPSMV RNA1 cDNA |
| CPSMR1-5389R | GGCCTCATGCATCTTTCTATGTG | PCR, sequencing of CPSMV RNA1 cDNA |
| CPSMR1-3022R | GTACCTGTACTGAGCCACATCAAA | PCR, sequencing of CPSMV RNA1 cDNA |
| CPSMR1-3251R | AGCGGCACGACCTTTGATGTCAA | PCR, sequencing of CPSMV RNA1 cDNA |
| CPSMR1-674F | CAGTTCGTTTCAGGACTTGCTGACTG | PCR, sequencing of CPSMV RNA1 cDNA |
| CPSMR1-5596R | GAGATTAGCCTGTGCCCAACCATGAG | PCR, sequencing of CPSMV RNA1 cDNA |
| CPSMR1-570F | TGCGTGGAGTTTATCTCAGC | Sequencing of CPSMV RNA1 cDNA |
| CPSMR1-1650F | TGCATGGAACGAGTGGTACT | Sequencing of CPSMV RNA1 cDNA |
| CPSMR1-2984R | TACTCTGTGCAACAACCGCT | Sequencing of CPSMV RNA1 cDNA |
| CPSMR1-5308F | TGTAGACTCCAATGACAATCTGG | Sequencing of CPSMV RNA1 cDNA |
| CPSMR1-3097F | GGAAAAGTGCAAGGCCAATTTCGT | Sequencing of CPSMV RNA1 cDNA |
| CPSMR1-1583F | CCTACAGTTTGTGGTTTGATCAA | Sequencing of CPSMV RNA1 cDNA |
| CPSMR1-2603R | TTGTGGGATCCACCAAATCTG | Sequencing of CPSMV RNA1 cDNA |
| CPSMR1-1448R | GCTTTAGCCACCCATCAACGTTTTCAGA | PCR, sequencing of CPSMV RNA1 cDNA |
| CPSMR1-1208F | GAGATGCTAGAGTCACTTGCTAAGAAC | PCR, sequencing of CPSMV RNA1 cDNA |
| CPSMR1-3230R | CAGTATAAATATCCACATACATTGGACTA | PCR, sequencing of CPSMV RNA1 cDNA |
| CPSMR1-2995F | CAGGTTTGATGTGGCTCAGTACAG | PCR, sequencing of CPSMV RNA1 cDNA |
| CPSMR1-5023R | CCTTTTGAGAAAGTCACAATCTTCCAA | PCR, sequencing of CPSMV RNA1 cDNA |
| CPSMR1-4722F | CATTGACTGTCATTTGCAACAGCAT | PCR, sequencing of CPSMV RNA1 cDNA |
| CPSMR1-802R | CATAGGACAGTACTCAACAAGCAAT | PCR, sequencing of CPSMV RNA1 cDNA |
| CPSMR1-1858F | TGGTGCTGTGTCTTCAGATCCTT | PCR, sequencing of CPSMV RNA1 cDNA |
| CPSMR1-3667F | GCAGGTAGAAATACCAGTTCAAG | PCR, sequencing of CPSMV RNA1 cDNA |
| CPSMR1-5356F | GTTTGTTGCCCCACATAGAAAGAT | PCR, sequencing of CPSMV RNA1 cDNA |
| CPSMR1-1419F | TTTCTGAAAACGTTGATGGGTGGCTAA | PCR, sequencing of CPSMV RNA1 cDNA |
| CPSMR1-4750R | GAAAATGCTGTTGCAAATGACAGTCAATG | PCR, sequencing of CPSMV RNA1 cDNA |
| CPSMR1-3194F | AGTCTCAATAGTCCAATGTATGTG | PCR, sequencing of CPSMV RNA1 cDNA |

**Table S1** (continued):

| **Name** | **Sequence (5’ to 3’)** | **Purpose** |
| --- | --- | --- |
| GA-CPSMR2-1Fb | TTCATTTGGAGAGGACCGGTTTATT AAAATTTTTCTAGGAAAATT | Generation of full-length CPSMV RNA2 cDNA with PCR |
| CPSMR2-BrgF2 | CTCGCAGATGCAAGATGTCCTACCC GGGAATCCCAGGCTTGACATCAAAG | Stepwise generation of CPSMV RNA2 full-length cDNA |
| CPSMR2-BrgR2 | CTTTGATGTCAAGCCTGGGATTCCC GGGTAGGACATCTTGCATCTGCGAG | Stepwise generation of CPSMV RNA2 full-length cDNA |
| CPSMR2-662F | ACCCAATGTGGATGAAATCTACGA | PCR & sequencing of CPSMV RNA2 cDNA |
| CPSMR2-631F | ATAGCCACAACTCGCAGATGCAAGAT | PCR & sequencing of CPSMV RNA2 cDNA |
| CPSMR2-3243R | GACCTTTGATGTCAAGCCTGGGATTC | PCR & sequencing of CPSMV RNA2 cDNA |
| CPSMR2-593F | ACAAAGTGGAATACCAGCAGAC | PCR & sequencing of CPSMV RNA2 cDNA |
| CPSMR2-3081F | TGGAGTAATCAAACCACATGGA | PCR & sequencing of CPSMV RNA2 cDNA |
| CPSMR2-3352R | TGCAAACCAAACTTACCTCTGC | PCR & sequencing of CPSMV RNA2 cDNA |
| CPSMR2-3006F | CCACATGCGTGGGAAAAGGAAT | PCR & sequencing of CPSMV RNA2 cDNA |
| CPSMR2-3126F | GCTTCTCAGTCCGCAATATTCG | PCR & sequencing of CPSMV RNA2 cDNA |
| CPSMR2-2066R | ATGCCAGTTTATGGTCATAAAGGT | PCR & sequencing of CPSMV RNA2 cDNA |
| CPSMR2-1679R | GAGAACTGTACCTGCAACTAATG | PCR & sequencing of CPSMV RNA2 cDNA |
| CPSMR2-1420F | TTCTAGTTTTCGATATATGATTTCTG GCAA | PCR of CPSMV RNA2 cDNA |
| CPSMR2-1450R | TTGCCAGAAATCATATATCGAAAACT AGAA | PCR of CPSMV RNA2 cDNA |
| CPSMR2-1159F | GAGATCTGGCGATGATGCATGTGAAT | PCR & sequencing of CPSMV RNA2 cDNA |
| CPSMR2-3160R | CGATAGAATATTTCGAATATTGCGGA | PCR & sequencing of CPSMV RNA2 cDNA |
| CPSMR2-2865F | TGTGGAACGCTGCATGTTAAAGTTGT | PCR & sequencing of CPSMV RNA2 cDNA |
| CPSMR2-758R | CTTTCCGAGCTTGGCAATACTTGT | PCR & sequencing of CPSMV RNA2 cDNA |
| CPSMR2-1834F | GTGGCATTCGAGGAAAAGCTTCTG | PCR & sequencing of CPSMV RNA2 cDNA |
| CPSMR2-2949R | GCATCACATACCCAAATGTCAC | PCR & sequencing of CPSMV RNA2 cDNA |
| NbACT-F2 | CAGCCACACTGTCCCAATTTATGAG | RT-PCR detection of NbACT mRNA |
| NbACT-R | CACCTTAATTTTCATACTGCTTGGA | RT-PCR detection of NbACT mRNA |
| NbPDS-450F | TGCATGGAAAGATGATGATGGAGAT | RT-PCR detection of NbPDS mRNA |
| NbPDS-979R | GTCTCTCAGGAGGGTTACCATCTAA | RT-PCR detection of NbPDS mRNA |
| GmACT4-F2 | TCAACCACTCGTCTGCGATAATG | RT-PCR detection of soybean actin 4 mRNA |
| GmACT4-R2 | CCAGCTTTTCCTTTACATCCCTTA | RT-PCR detection of soybean actin 4 mRNA |
| GmPDS1-938F | CCATATGTTGAGGCTCAAGATG | RT-PCR detection of soybean PDS1 mRNA |
| GmPDS1-1464R | AGGTGATCATATGTGTTCTTCAG | RT-PCR detection of soybean PDS1 mRNA |
