# Supplementary material for: A cowpea severe mosaic virus-based vector simplifies virus-induced gene silencing and foreign protein expression in soybean
Source: Plant Methods. 2022 Oct 28;18:116. doi: 10.1186/s13007-022-00950-7 (PMC9617382; doi:10.1186/s13007-022-00950-7)
Supplement: Supplementary file 2 — Additional file 2: Table S2. Sequences of gBlock fragments used in this study. [file 13007_2022_950_MOESM2_ESM.docx]

**Table S2**. Sequences of gBlock fragments used in this study.

Letters in red: introns; underlined: restriction enzyme (RE) sites used for inserting the gBlock fragments into CPSMV RNA1 or 2 cDNA.

| **Name (bp)** | **Sequence** | **RE sites** |
| --- | --- | --- |
| CPSMR1-327Intron (675 bp)  (Intron a) | TTCAAAATCACTTTTTCTCTCTTAAGCTACACAAGTTTAACTAAAAGCCTAGAACGAACTTTTTATTGGACGCATTAAACTACAATATGAAGTTCTTTGCTGGGCAAACTGTTATGAATGTGCTGCAACATGTTTCCTCTCCCACCACCAATTTAAGGTTTACTACCATGCTGTCTTGTGGGTTTAAGAATTCATCTGGTATCCTGCTTATTAATATGTTGTTTATGTTTTTCTTGTAGGTTGTTATCTTATTGCAATCTTAAAAAGGAAGAGGATGGTAAGATGATGCTGGCTATTAAGGAGCAGAGGCACCGACGGTTGTTGACACTGTCATATGGTGCTATGTGTTTTCAATTCTCAAATTCAGTAGGGGATGAGGGCATAGAAGTGGATGATGATGAGTTGATGTTTGAAATATTTGATGCATTGCTGCGCACAAAGATTTCAAACTCAAAGGGCATGACGCACTTATACAGCTGGATGCGTGGAGTTTATCTCAGCACATTCAAAGTAGAGGTGCAGTGTGATGATTACAACTCAAATCTGCTGGAGAAGGATTTGGCTGGAGAAGCTCAGGGTCTTTCACAGTTCGTTTCAGGACTTGCTGACTGGATTCCCAGTCGTGTAAAGACCTTGGCAGGATATGCCGCTGAGGGCATAATTGAGGCCTTTAAG | AflII, BbvCi |
| CPSMR1-604Intron (675 bp)  (Intron b) | TTCAAAATCACTTTTTCTCTCTTAAGCTACACAAGTTTAACTAAAAGCCTAGAACGAACTTTTTATTGGACGCATTAAACTACAATATGAAGTTCTTTGCTGGGCAAACTGTTATGAATGTGCTGCAACATGTTTCCTCTCCCACCACCAATTTAAGGTTGTTATCTTATTGCAATCTTAAAAAGGAAGAGGATGGTAAGATGATGCTGGCTATTAAGGAGCAGAGGCACCGACGGTTGTTGACACTGTCATATGGTGCTATGTGTTTTCAATTCTCAAATTCAGTAGGGGATGAGGGCATAGAAGTGGATGATGATGAGTTGATGTTTGAAATATTTGATGCATTGCTGCGCACAAAGATTTCAAACTCAAAGGGCATGACGCACTTATACAGCTGGATGCGTGGAGTTTATCTCAGCACATTCAAAGTAGAGGTTTACTACCATGCTGTCTTGTGGGTTTAAGAATTCATCTGGTATCCTGCTTATTAATATGTTGTTTATGTTTTTCTTGTAGGTGCAGTGTGATGATTACAACTCAAATCTGCTGGAGAAGGATTTGGCTGGAGAAGCTCAGGGTCTTTCACAGTTCGTTTCAGGACTTGCTGACTGGATTCCCAGTCGTGTAAAGACCTTGGCAGGATATGCCGCTGAGGGCATAATTGAGGCCTTTAAG | AflII, BbvCi |
| CPSMR1-Tin  (1731 bp)  (Introns #1&2) | ACTGTGGCAAAGGATGGTACTGTACACTGTTGGGCAGAATCTGGGAAGAACTCATTCTAGTTGGTATGAGGGCATCAAGGACAAGTGCATGCTTGCACTATCAAAAGCATACTCAACTGAGATCAAGGATTGGCCTGTAGCACTCAAAATTGTTGTTGGAGTGATACTGGCTACTGTAGCAGGTTTACTACCATGCTGTCTTGTGGGTTTAACAATTCATCTGGTATCCTGCTTATTAATATGTTGTTTATGTTTTTCTTGTAGGTAAGGCATTTTGGAGGTTCTATGCCTCAATGGCAGATGCAGGCAATGGTGGACACTTTGTGGGAGCCGTTGCTTCCGCATTTGCAGGAAGTCAAGCGGTTGTTGCACAGAGTAGGAAGCCCAACAGGTTTGATGTGGCTCAGTACAGGTACCGAAACATACCTCTAAGGAAGAGAAATTGGGCAGAAGGGCAAATGAGTCTGGATCAGTCCACAATGCTCATAATGGAAAAGTGCAAGGCCAATTTCGTCTTTAGCAACATTAGCTGTCAGATAGTTATGTTGCCTGGGCGACAATTCTTGTGCTACAAACATGTGTTTGCTAGTCTCAATAGTCCAATGTATGTGGATATTTATACTGCCAACAAGAAGTATAAACTCTATTACAAACCTCAGAATAGGGTATACTTTGAGACTGATAGTGAGATCATGCTATACAAGGATGCCAGTTTGGAAGACATACCTGCCAGCTGCTGGGATCTTTTTTGTTTTGATGCGGAAAAAAGTCTGCCACGAGGTAGTTTCCCAGCAGAAATCCTCTCGTGCAAACTAGATCGGACAACGAATCAACATATCCCGGAGTGGGCCGACATCTCAGCTCGTACTGTCAATCAAAAACTGGACGTGGAATTTGGGGAGTACCAAACCATCTTTTATTCCTATCTCCAGTATGATGTACCCACAAAAGCTGAAGATTGTGGTTCCCTAATAATAGCAACCATTGATGGTAGGAAAAAGATAATAGGGATCCACACTGCTGGACGGGCAAATAGGAGTGGTTTTGCAAGTTATATGCCGCAGGTAGAAATACCAGTTCAAGCACAAGCAGCGGAAAAGTTCTTTGATTTTCTTGAGAAaGaACAACATGTTACTGAGGGCATTGGAAAGGTGGGAAATCTCAAGAAAGGAGTCTGGGTTCCATTACCCACTAAGACCAATCTTGTGGAAACACCAAAAGAGTGGCATCTGGGCACTGAGAAAACAAAAGAACCAAGTATTCTCAGCAGTACGGATTTAAGGCTCGGTGATAAGCAGTATGATCCCTTTGTTGGAGGAATACAGAAGTACGCCGAACCAATGGGAATTCTAGATGATGAGGTGCTCCGGCATGTGGCAACAGACATAGTTGAAGAATGGTTTGACTGTGTAGACCCTCAAGAAGATACTTTTGAGGAAGTTGACCTGCAGGTTGCTATCAATGGTCTTGAAGGAATGGAATACATGGAAAGAGTTCCTATGGCAACATCTGAAGGCTTCCCACACATTTTGACAAGGAAAAGTGGGGAAAAAGGCAAAGGTTAGTGAAGTTGTACCATTTATTTAGCTTCAAATGAGGGACCAGGCTTTACATTGATAAATTCAACATCTTTCTCTATTGTTGCATGATCAGGTAGGTTTGTATATGGGGATGGAGAAATTTTTGATCTGATCCCGGGTACATCTGTACATGAGGCATATCTGACACTGG | Bsp1407I |
| CPSMR1-TRIN  (1812 bp)  (Introns #1,2,3) | CTGTGGCAAAGGATGGTACTGTACACTGTTGGGCAGAATCTGGGAAGAACTCATTCTAGTTGGTATGAGGGCATCAAGGACAAGTGTATGCTTGCACTATCAAAAGCATACTCAACTGAGATCAAGGATTGGCCTGTAGCACTCAAAATTGTTGTTGGAGTGATACTGGCTACTGTAGCAGGTTTACTACCATGCTGTCTTGTGGGTTTAACAATTCATCTGGTATCCTGCTTATTAATATGTTGTTTATGTTTTTCTTGTAGGTAAGGCATTTTGGAGGTTCTATGCCTCAATGGCAGATGCAGGCAATGGTGGACACTTTGTGGGAGCCGTTGCTTCCGCATTTGCAGGAAGTCAAGCGGTTGTTGCACAGAGTAGGAAGCCCAACAGGTTTGATGTGGCTCAGTACAGATACCGAAACATACCTCTAAGGAAGAGAAATTGGGCAGAAGGGCAAATGAGTCTGGATCAGTCCACAATGCTCATAATGGAAAAGTGCAAGGCCAATTTCGTCTTTAGCAACATTAGCTGTCAGATAGTTATGTTGCCTGGGCGACAATTCTTGTGCTACAAACATGTGTTTGCTAGTCTCAATAGTCCAATGTATGTGGATATTTATACTGCCAACAAGAAGTATAAACTCTATTACAAACCTCAGAATAGGGTATACTTTGAGACTGATAGTGAGATCATGCTATACAAGGATGCCAGTTTGGAAGACATACCTGCCAGCTGCTGGGATCTTTTTTGTTTTGATGCGGAAAAAAGTCTGCCACGAGGTATGTGGATGAGTTCTTGTGTATTTTATCAGGAGATTGTAAATTTAGTGCAATCTTGAATTATCGATTCTATTACTCCACAGGTAGTTTCCCAGCAGAAATCCTCTCGTGCAAACTAGATCGGACAACGAATCAACATATCCCGGAGTGGGCCGACATCTCAGCTCGTACTGTCAATCAAAAACTGGACGTGGAATTTGGGGAGTACCAAACCATCTTTTATTCCTATCTCCAGTATGATGTACCCACAAAAGCTGAAGATTGTGGTTCCCTAATAATAGCAACCATTGATGGTAGGAAAAAGATAATAGGCATCCACACTGCTGGACGGGCAAATAGGAGTGGTTTTGCAAGTTATATGCCGCAGGTAGAAATACCAGTTCAAGCACAAGCAGCGGAAAAGTTCTTTGATTTTCTTGAGAAaGaACAACATGTTACTGAGGGCATTGGAAAGGTGGGAAATCTCAAGAAAGGAGTCTGGGTTCCATTACCCACTAAGACCAATCTTGTGGAAACACCAAAAGAGTGGCATCTGGGCACTGAGAAAACAAAAGAACCAAGTATTCTCAGCAGTACGGATTTAAGGCTCGGTGATAAGCAGTATGATCCCTTTGTTGGAGGAATACAGAAGTACGCCGAACCAATGGGAATACTAGATGATGAGGTGCTCCGGCATGTGGCAACAGACATAGTTGAAGAATGGTTTGACTGTGTAGACCCTCAAGAAGATACTTTTGAGGAAGTTGACCTACAGGTTGCTATCAATGGTCTTGAAGGAATGGAATACATGGAAAGAGTTCCTATGGCAACATCTGAAGGCTTCCCACACATTTTGACAAGGAAAAGTGGGGAAAAAGGCAAAGGTTAGTGAAGTTGTACCATTTATTTAGCTTCAAATGAGGGACCAGGCTTTACATTGATAAATTCAACATCTTTCTCTATTGTTGCATGATCAGGTAGGTTTGTATATGGGGATGGAGAAATTTTTGATCTGATCCCGGGTACATCAGTACATGAGGCATATCTGACACTG | Bsp1407I  (2^nd^ site lost upon inserting gBlock) |
| CPSMR1-QUIN  (953 bp)  (Intron #4) | TGTTTATGCACATGGGGCTTCTAGAGTTGGTAAAACAATGGTGATCAACAGACTCATTGAAGATTTTCGCAAAGAGTTGGAACTTGGAGAGGACTGTGTGTATCCACGAAATGTGGTAGATGACTACTGGAGTGGGTACAAAAGACAACCTATTGTTGTCATTGATGATTTTGGTGCTGTGTCTTCAGATCCTTCTGCAGAAGCTCAATTAATTCCATTGATCTCTAGTGCTCCCTATCCCCTTAACATGGCTGATCTCTCTGAGAAGGGAATGCACTTTGATTCAGCTATCGTCATGTGCTCATCCAATTTCATTGAGTGTTCACCAGAAAGCAAGGTACTTTCTCAAAATCAAAAGAAACTGTAGTGTAACAAATTTCTTCACTTGATGTGATGTATATATATCTTTAGCTATCTAGTGTTCATATATGTAACTCGTTGTTCAAGAACTTCTTTGTGTAATTCATCCGCAGGTGCGTGACGAAATGGCATTCAGAAACAGACGACATGTGCTCTTCACTGTCTCACTTGACCCTAATATACCATATGATGGTGATGATATCACAAAGAATCAAATATATGAAATCAAAACTTGGTTTCATGATTCGTATCATGTTGAAGCAACTTTCACATCATATGGGGACTTGCTGGCATATTGCAAAAACAAGTGGGTGGAGCACAATACTGAGCAAGAGGCCAACTTGAAGCAACTTGGAGTTAAAAAGGAGAGCGTTGCATTTCAGCAGTTTCGTTCCATTCTTGATTTGGCAGTCTTTGTCAATCAAGATGCGGAGAATTTCAAGCAAAGGCTGGAGACGCCAGATGGTAGGTGCCACTTTGTGTCATGTTATGATAAGAGTGGTATACTCAGGCACTATACTATTGATGCAACTGGAGATGTGCAAGAAATGGAAAAGGTTGATTCCTCTCTAGATGACATCCTATTGGAAAAA | XbaI |
| FZVec-ML-NbPDS  (934 bp)  Bold blue: duplicated processing sites;  Dark red: altered to minimize recombination;  Light red: new Eco72I sites;  Simple blue: NbPDS insert | TTTGAAGGGCAACCAGTGCTTAAGAGAATCTCATCTACCAAAGCTATTTTCTCTAAAGGTTCTAGTTTTCGATATATGATTTCTGGCAAAAAAGAGCACAAGATTGACAAGCCAAGGCTAGAAGAAGATGGTAGTAAGAGTTACATTGATGGTTTACAAGATACCTTTGACACGACTCATGCTACTCTGCAA**TCAGGTGCAGACCTGTTTAAGAGGAACCTAGACGATGTCTCGACTATCTCAGATACGATGCACGTG**CAAGGTGTGCCTGATAGGGTGACAGATGAGGTGTTCATTGCCATGTCAAAGGCACTTAACTTCATAAACCCTGACGAGCTTTCGATGCAGTGCATTTTGATTGCTTTGAACAGATTTCTTCAGGAGAAACATGGTTCAAAAATGGCCTTTTTAGATGGTAACCCTCCTGAGAGACTTTGCATGCCGATTGTGGAACATATTGAGTCAAAAGGTGGCCAAGTCAGACTAAACTCACGAATAAAAAAGATCGAGCTGAATGAGGATGGAAGTGTCAAATGTTTTATACTGAATAATGGCAGT**CACGTGACGTTCGATACCACACACGCAACCCTACAG**TCTGGGGCTGATCTATTCAAACGAAATTTGGATGATGTAAGCACCATTTCGGACACCATGCTTGGGGCCATGATTGGACAAACCAAGGTGGTGATTCCAAAAACATTAGTTGCAGGTACAGTTCTCAAAAGTGGACCGCTTTCAGATGTGATGCAGCAGGGATCATTCCGATCAACAATAGCATTGCAAAGAACACATATAATAACTGGAAAAATACATGTTGTTGCGATGCTTGAAACTGCTGTAAATACAGGACTGGGATTGGCCATTTGTTTCAATAGTGGCATTCGAGGAAAAGCTTCTGCAGACATCTATGC**A**ACGTGTTCACAAGATGCCATGA | AflII, Eco72I (lost upon inserting gBlock) |
